# Maternal Body Mass Index and Anovaginal Distance in Active Phase of Term Labor

**DOI:** 10.1155/2018/1532949

**Published:** 2018-03-07

**Authors:** Linda Hjertberg, Eva Uustal, Sofia Pihl, Marie Blomberg

**Affiliations:** Department of Obstetrics and Gynecology and Department of Clinical and Experimental Medicine, Linköping University, S-581 85 Linköping, Sweden

## Abstract

**Introduction:**

To evaluate if there was a difference in the anovaginal distance (AVD) measured by transperineal ultrasound between obese and normal weight women.

**Material and Methods:**

A prospective observational study including 207 primiparous women at term in first stage of labor. Transperineal ultrasound with a vaginal probe was used to measure the AVD. Maternal, pregnancy, and delivery characteristics potentially associated with perineal thickness were extracted from woman's medical records. The participants were divided into three BMI groups based on maternal weight in early pregnancy: normal weight (BMI < 25), overweight (BMI 25–29.9), and obesity (BMI ≥ 30). Obese and overweight women were compared with normal weight women regarding the AVD.

**Results:**

The mean AVD was 24.3, 24.9, and 27.0 mm in the normal weight, overweight, and obesity group, respectively. There were no group differences in background characteristics. The AVD was significantly longer in obese women compared with normal weight women (*p* = 0.018).

**Conclusions:**

The observed longer AVD in obese women might be protective of the anal sphincter complex, explaining lower rates of anal sphincter injuries in this group. Further studies are indicated to evaluate whether the length of the AVD plays a role in the risk assessment of obstetric anal sphincter injury. The trial is registered in ClinicalTrials.gov and the trial registration ID is NCT03149965.

## 1. Introduction

There are indications that diagnosed obstetric anal sphincter injuries (OASIs) occur more seldom in obese women than in normal weight women, although results are not fully consistent [1–3]. A possible explanation could be that the OASIs among obese women are more difficult to detect and is therefore undiagnosed to a larger extent. Another possibility could be that maternal obesity is a protective factor for severe perineal damage during vaginal delivery due to a longer or thicker perineum.

A short/thin perineal body has been shown to be associated with a higher degree of perineal/anal laceration during vaginal delivery [4, 5]. The perineal body has in former studies [6] been defined as the fibromuscular mass located between the anal canal and posterior vaginal wall and represents a central stabilizing point of the pelvic floor. It serves as the medial insertion point of the puborectalis, bulbospongiosus, external anal sphincter, internal anal sphincter, superficial and deep transverse perineal muscles, and the rectovaginal fascia cranially [7]. Different methods to measure the perineal body height or size are described: palpation, inspection, Magnetic Resonance Imaging [8], and endoanal, translabial, and transperineal ultrasound [9–12].

We hypothesized that the anovaginal distance (AVD) in term pregnancy, defined as the distance between the anal mucosa and the vaginal wall at the middle level of the anal canal, is longer in obese women compared to normal weight women.

This study aimed to evaluate if there was a difference in AVD measured by transperineal ultrasound between obese and normal weight women in active phase of labor at term pregnancy.

## 2. Material and Methods

This is a prospective observational study including primiparous women in active phase of labor at term pregnancy on admission at the delivery ward at Linköping University hospital. The study period was between October 2014 and March 2016. Term pregnancy was defined as ≥37–≤42 gestational weeks. The women had to be in active labor according to the Swedish definition; two out of three of the following criteria must be present: painful contractions (two to three contractions in every ten minutes), cervix shortened and dilated > one centimeter (cm), and/or rupture of membranes. The maximum cervix dilatation allowed for inclusion was seven cm to avoid any potential effects on the perineal floor due to circumstances linked to second stage of labor. Further the participant had to be proficient in the Swedish language and aged ≥18 years. All participants were given written and verbal information of the study. The verbal consent to participate was documented in the women's medical record.

**Figure 1:**
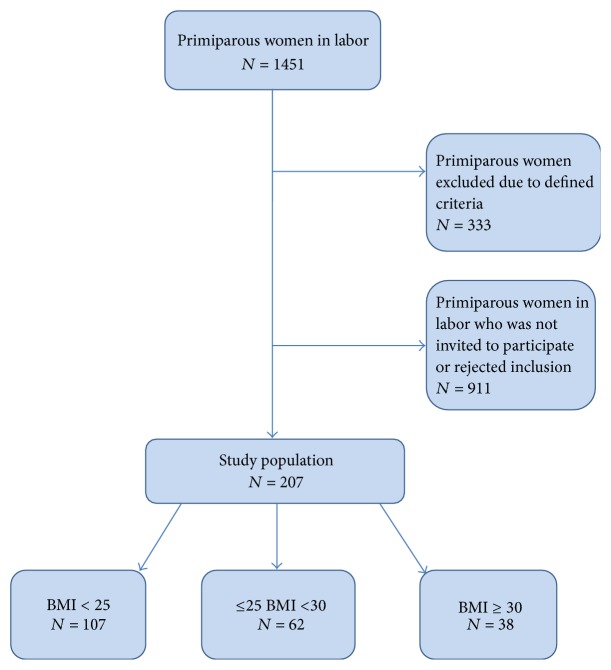
Flowchart of the study population.

A flowchart of the study population is presented in Figure 1. A total number of 207 women were included in the study. There was no information available about whether the women declined participation or if they were not invited to participate. 333 women were excluded due to prematurity, cervix dilatation > seven cm when the women were signed in at the delivery ward, being younger than 18 years, or not speaking satisfactory Swedish language. After the women provided the informed consent, the AVD was measured with transperineal ultrasound.

**Figure 2:**
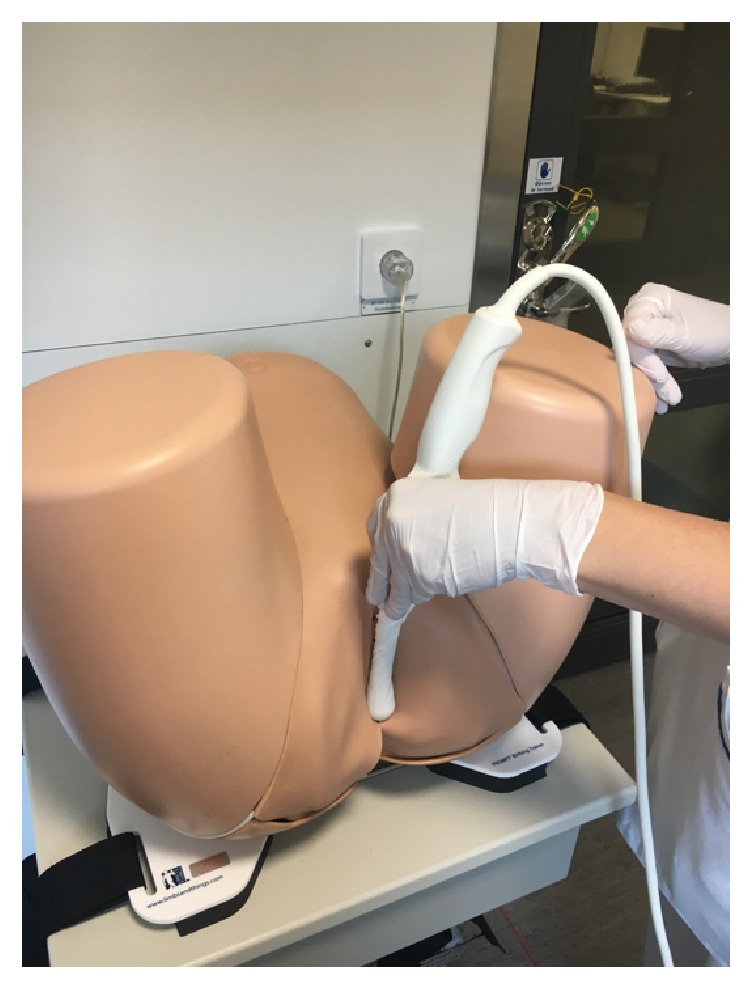
Transperineal ultrasound method to determine the anovaginal distance.

**Figure 3:**
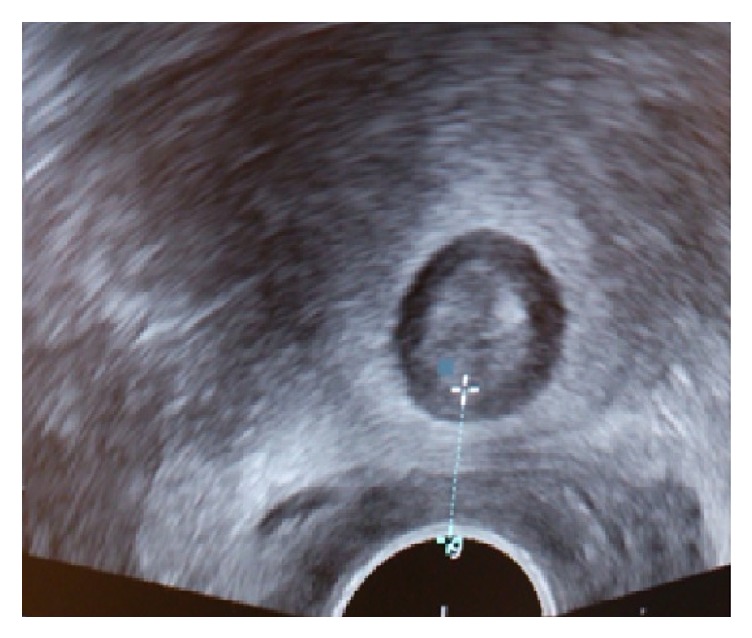
The anovaginal distance is shown from the internal sphincter mucosa to the posterior vaginal wall.

The standardized method of measuring AVD consisted of placing the vaginal probe at a right angle to the posterior vaginal distal wall and in a transversal scanning plane (Figure 2). All examinations were done with the woman in the lithotomy position. The internal anal sphincter was detected as a low-echogenic ring when the probe was moved cranially from the distal anal canal to the mid anal canal. The AVD in this study was measured and defined as the distance between the anal mucosa and the vaginal wall at the middle level of the anal canal (Figure 3). Transperineal ultrasound of the AVD has been evaluated and showed a short learning period for examiners with earlier ultrasound experience and a high interobserver agreement. With an accepted difference of ≤5 mm interobserver variation, the weighted kappa-coefficient was 0.87 (*p* ≤ 0.001) with an agreement of 92.5%, classified as almost perfect agreement [13]. The measured AVD, in millimeters (mm), was documented in the women's medical record under a specific keyword named “anovaginal distance.”

The examiners had different proficiency in vaginal ultrasound. As a minimum training to be allowed to include women in the study, the midwives and the obstetricians were trained individually in a defined education program. The education program was based on comeasuring the AVD with one of two experienced experts of AVD measurement. All examiners had to perform comeasurements in at least five women in term pregnancy. Each woman was, after informed consent, measured three times by both the expert and the examiner at education. These women were not included in the present study. The measured AVD values were not shared by the expert and the examiner. The AVD values were compared afterwards, outside the delivery room. In order to be given permission to independently measure the AVD, the interobserver variability between the examiner and the expert had to be at maximum +/− five mm in five women. There was no specific requirement of intraobserver variability. Proficiency was achieved among all examiners

Maternal weight and maternal height measured in early pregnancy (gestational week 10–12) were extracted from the digitalized medical records for every participant and Body Mass Index (BMI) was calculated. The study population was then divided into three BMI classes: normal weight (BMI < 25), overweight (BMI 25–29.9), and obesity (BMI ≥ 30).

A number of maternal, pregnancy, and delivery characteristics that potentially could affect the thickness of the pelvic floor in term pregnancy were extracted from the digitalized medical records for every participant and manually registered in an anonymous research database together with maternal BMI and measured AVD. Maternal age and ethnicity were two putative factors that could potentially affect the thickness of the perineum. A third one was low or extensive gestational weight gain. Gestational weight gain was defined as the difference between the registered weight in kilograms (kg) at the first antenatal visit to the antenatal care center and the registered weight in term pregnancy at the delivery ward. If there was no registered weight at the delivery ward, the last registered weight at the antenatal care center (between gestational weeks 37–42) was used to calculate gestational weight gain. The actual stage of labor at the time of the AVD measurement could possibly interfere with the thickness of the perineum and was therefore extracted from medical records and included in the database. The present stage of labor was defined in two ways: as a cervical dilatation more or less than five centimeters and as a position of the fetal presenting part above or below the ischial spine.

The number of women included was based on a power calculation where the clinically relevant difference in AVD between the BMI groups was set to be 10 mm and significance level was equal to five percent. We stipulated that a difference of five mm in the AVD could be of clinical relevance. The sample size in each BMI group was calculated to be at least 30 to reach a power of 80% to detect five mm difference in the AVD. Sample size determination was based on a two-sample independent *T*-test.

All primiparous women that fulfilled the inclusion criteria during the study period were invited to participate. Hence, we did not take any concern of their BMI when inviting them to the study. The inclusion stopped when there were more than 30 participants in the obesity group, just as the power analysis was set for.

Statistical analyses were performed in order to compare maternal, pregnancy, and delivery characteristics between different BMI groups using one-way ANOVA for continuous variables and *X*^2^-test for categorical variables. Post hoc tests were adjusted by Tukey's method. A *p* value of <0.05 was considered significant. Statistical analyses were performed using the Statistical Package for the Social Sciences (IBM SPSS Statistics, Chicago, IL, USA; version 22).

### 2.1. Ethical Approval

The Regional Ethical Review Board in Linköping has approved the study (Dnr 2014/245-31).

## 3. Results

The study population consisted of 207 primiparous women in active phase of labor at term pregnancy. They were distributed as follows: normal weight (BMI < 25) *n* = 107; overweight (BMI 25–29.9) *n* = 62; and obese (BMI ≥ 30) *n* = 38. Maternal and obstetrical characteristics of the study population are presented in Table 1. There was no significant difference between the three BMI groups concerning age (mean age in year), smoking status, ethnicity, gestational weight gain (mean value), cervix dilatation status (<5 cm or >5 cm), or fetal presenting part (above the ischial spines or below the ischial spines). Since there was no statistically significant difference over the BMI strata in any of these factors, no further adjustments analyzing AVD in relation to maternal BMI were performed.

The mean value of the AVD in millimeters and the 95% confidence interval for each BMI group are presented in Table 2. There was a statistically significant difference in the mean AVD over the BMI groups (*p* = 0.024). Obese primiparous women had a significantly longer AVD compared to normal weight primiparous women (*p* = 0.018).

The mean value of the AVD in all studied primiparous women in early labor (*N* = 207) was 25.0 mm (13–44 mm).

## 4. Discussion

This prospective observational study showed that primiparous obese pregnant women in active phase of labor at term pregnancy had a significantly longer AVD, measured by transperineal ultrasound, compared to normal weight women. This means that obese women have a longer distance between the anal mucosa and the vaginal wall at the middle level of the anal canal than their normal weight counterparts. To our knowledge, evaluation of the perineal thickness by transperineal ultrasound in different BMI groups in active phase of labor has not been done before. The tissue components in the perineal floor that contributes to the longer AVD could only be speculated upon: is it an increased muscle mass due to higher pressure on the pelvic floor in obese women or is it an increased fat mass volume? The finding that a difference in AVD existed has two major implications. First it warrants further evaluation of the tissue components located between the anal canal and the vaginal wall over the maternal BMI strata and secondly it indicates that the length of AVD could play a role in the risk assessment of OASI as obese women (with longer AVD) seem to have a decreased risk [1] of this unwanted complication of vaginal delivery.

To our knowledge there are no other studies assessing the anovaginal distance or the perineal tissues in full term pregnancy using transperineal ultrasound. Hence, the perineal area has been evaluated in an obstetric purpose with other methods both during pregnancy but more often in the postpartum period. The golden standard to assess the perineal tissues including the anal sphincter complex is endoanal ultrasound [9, 14, 15]. Mayooran et al. evaluated the perineal body and the anal sphincter complex by endoanal ultrasound antenatally in gestational weeks 38–41 [15]. They measured the thickness of the puborectalis muscle, the external anal sphincter, the intersphincteric space, the subcutaneous component, and the internal anal sphincter separately at different levels in the anal canal. No additive measure of all components was presented making comparison with the present study difficult, nor did they divide the study population into BMI groups. Another major difference was that these measurements were performed with endoanal ultrasound, a technique affecting the perineal tissues in a different manner compared to transperineal ultrasound. There are no available comparative studies between transperineal ultrasound and endoanal ultrasound at term pregnancy.

The Pelvic Organ Prolapse Quantification System (POP-Q) method has been used to establish the perineal skin length in term pregnancy, during second stage of labor, and postpartum [16, 17]. The main result was that the perineal body measured with the POP-Q system increased during labor but the length during labor did not correlate with the degree of vaginal laceration. The POP-Q method measures the skin length of the perineum and therefore comparison with results from the present study measuring the distance from the mid anal canal to the posterior vaginal wall is tricky. Translabial pelvic ultrasound has also been used to determine the changes of the perineal body and the anorectal junction mobility in term pregnancy compared with the postpartum period [12]. They concluded that this methodology of measuring the perineal tissue had certain technical limitations, mainly due to differences in width of the levator hiatus and degree of pelvic organ prolapse between the included women which could have affected the transducer movements.

A number of studies have evaluated the perineal area in relation to obstetrical vaginal lacerations and anal sphincter injury most of them using endoanal ultrasound. There are however a few using transperineal ultrasound as equal method to the present study. Transperineal ultrasound, both two- and three-dimensional, has been used for assessment of anal sphincter injuries and their repair in the postpartum period [10, 18]. Ozyurt et al. used transperineal ultrasound with a vaginal ultrasound probe before hospital discharge, after vaginal delivery in order to evaluate the incidence of OASIs, and they found a significant difference in the maternal mean perineal body distance with a thinner perineum in cases with OASI [19]; however, our study was not powered to relate the length of AVD to the occurrence of OASI.

The present study has certain strengths and limitations. One limitation could be the number of missing potential study subjects. Inclusion or not was determined by the actual workload at the unit. We have no ethical permit to evaluate data on women who were not informed about the study or who declined to participate. The proportion of women in the study population with obesity was 17% (38/207) which is in accordance with the proportion of women with obesity in the general pregnant population. We have therefore no reason to suspect selection bias based on BMI. It is possible that the midwives and the obstetricians measurement of the AVD could have been influenced by being aware of the woman's BMI. At the time of the study there was no knowledge among the examiners about a potential association between the AVD and maternal BMI. Another shortcoming is that the use of transperineal ultrasound with a vaginal probe to measure AVD has only been evaluated in nonpregnant women [12]. Hence, there is no reason to suspect that the interobserver or intraobserver variability would increase by pregnancy. The method of transperineal ultrasound with a vaginal probe directly after childbirth was found to be painless and well tolerated by participating women.

## 5. Conclusions

There was a significantly longer AVD measured by transperineal ultrasound in obese pregnant primiparous women in active phase of labor compared to their normal weight counterparts. This longer AVD might be protective of the anal sphincter complex during second stage of labor, as lower rates of OASIs have been observed in the obese group. Now further studies are indicated to evaluate whether the length of the AVD plays a role in the overall risk assessment of OASI.

## Figures and Tables

**Table 1:** Maternal and obstetrical characteristics of the study population.

	BMI < 25 *n* = 107	25 ≤ BMI < 30 *n* = 62	BMI ≥ 30 *n* = 38	*p* value
Age mean (SD)	28.5 (4.0)	29.7 (5.7)	27.4 (5.2)	0.06
Weight gain during pregnancy (kg) mean (SD)	13.7 (4.2)	14.5 (6.5)	14.2 (6.6)	0.66
Gestational weeks mean (SD)	40.4 (1.2)	40.5 (1.2)	40.5 (1.4)	0.88
*Ethnicity n* (%)				0.82
Swedish	101 (94.4)	58 (93.5)	35 (92.1)	
Not Swedish	6 (5.6)	4 (6.5)	3 (7.9)	
*Cervical dilatation* (cm) *n* (%)				0.51
≤5	69 (64.4)	39 (62.9)	28 (73.7)	
>5	38 (35.5)	23 (37.1)	10 (23.6)	
*Presenting fetal part n* (%)				0.13^*∗*^
Above the ischial spines *n* (%)	106 (99.1)	59 (95.2)	38 (100)	
Below the ischial spines *n* (%)	1 (0.9)	3 (4.8)	0	

^*∗*^Assumption for Chi^2^ test not fulfilled.

**Table 2:** The mean value and 95% confidence interval of the anovaginal distance (millimeter) measured by transperineal ultrasound in normal weight, overweight, and obese term pregnant women in early active labor.

	*N*	Mean AVD (mm)	95.0% Lower CI for Mean AVD (mm)	95.0% Upper CI for Mean AVD (mm)	*p* value
BMI groups					0.024
Normal weight (BMI < 25)	107	24.3	23.3	25.3	
Overweight (BMI 25–29.9)	62	24.9	23.7	26.0	
Obesity (BMI ≥ 30)	38	27.0	25.0	28.9	0.018^a^

^a^Compared to AVD in normal weight women (BMI < 25); AVD = anovaginal distance; BMI = body mass index; CI = confidence interval.
